# Comment on “Prevalence of subhealth status and its effects on mental health and smartphone addiction: a cross-sectional study among Chinese medical students”

**DOI:** 10.1590/1806-9282.20220553

**Published:** 2022-08-19

**Authors:** Zixuan Zhou, Cuiping Li

**Affiliations:** 1 Taizhou University, School of Medicine – Zhejiang, China.

Dear editor,

We are pleased to read an article entitled “Prevalence of subhealth status and its effects on mental health and smartphone addiction: a cross-sectional study among Chinese medical students” by Zhang et al.^1^. The findings of this study suggest that anxiety, depression, and smartphone addiction among Chinese medical students are associated with subhealth status. This study is of great significance in the prevention and treatment of the physical and mental health of medical students. However, in our opinion, there are some questions that are still unanswered and worth discussing.

The main problem with this study is that Suboptimal Health Status Questionnaires-25 can only be used to assess suboptimal health status but not to diagnose suboptimal health status. Suboptimal health was defined in this study by a total subhealth status score ≥35, which is obviously subjective. Clearly, such diagnostic criteria for suboptimal health are not widely accepted by scientists. In fact, there are clear diagnostic criteria for suboptimal health^2^. According to the definition of subhealth, subjects with mental subhealth, overweight, prehypertension, pre-diabetes, serum blood lipids (triglycerides or total cholesterol) above the borderline high level, renal subhealth, hepatic subhealth, or thiobarbituric acid-reactive substances ≥5.09 μmol/L (based on the reference range of 95%) were categorized as subhealthy^2^.

Another problem is that this study is a non-probabilistic sample (web-based questionnaire). Thus, the sample is not representative. The limitations of the article include the non-probability sampling technique and sampling structure that is limited to a single university. In addition, there are many factors that affect subhealth. For example, the novel coronavirus disease 2019 (COVID-19) epidemic can also have deleterious consequences on depression and anxiety of college students^3^.

## CONCLUSION

The Suboptimal Health Status Questionnaires-25 can only be used to assess suboptimal health status but not to diagnose suboptimal health status. In addition, the causal relationship between subhealth and anxiety and depression needs to be further investigated.

## DATA AVAILABILITY

The data sets generated and analyzed during the current study are available from the corresponding author on reasonable request.
